# Benign osteoblastoma of the mandible in a 12-year-old female: A case report

**DOI:** 10.3892/ol.2014.2593

**Published:** 2014-10-09

**Authors:** KONSTANTINE MARDALEISHVILI, ZURAB KAKABADZE, AVTANDIL MACHAVARIANI, TEIMURAZ GRDZELIDZE, ANNA KAKABADZE, NATIA SUKHITASHVILI, TAMAR KURASHVILI, NESTAN SHONIA, GIORGI MENABDE, IVANE ABIATARI

**Affiliations:** 1 Department of Oncology, Tbilisi State Medical University, Tbilisi 0177, Germany; 2 Department of Clinical Anatomy, Tbilisi State Medical University, Tbilisi 0177, Germany; 3 Institute of Medical Research, Ilia State University, Tbilisi 0162, Georgia, Germany; 4 Department of Surgery, University of Technology Munich, Munich, Bavaria 81675, Germany

**Keywords:** bone tumor, osteoblastoma, mandible

## Abstract

Benign osteoblastoma refers to a benign tumor of the bone. Osteoblastoma most commonly affects the vertebrae and long tubular bones, however, in rare cases is observed in the facial bones. The current study presents the case of a 12-year-old female patient with a tumor in the mandibular body. Radiological imaging revealed a lesion with regular contours. The lesion was radically resected and histological analysis of the specimen demonstrated features that are typical of a benign osteoblastoma. The consequential defects of the jaw were reconstructed using titanium implants and autologous bone transplantation. The patient remains disease free subsequent to a five-month follow-up period. The aim of the present report is to present a rare case of benign osteoblastoma of the mandible. This study demonstrated that correct diagnosis and complete surgical excision are important to reduce the risk of recurrence of a benign osteoblastoma.

## Introduction

Benign osteoblastoma is an osteoid and bone-forming benign tumor that rarely occurs in the facial bones and was first described by Jaffe and Mayer in 1932 (1). The current term, benign osteoblastoma was proposed by Jaffe and Lichtenstein in two different reports in 1956 (2,3). Benign osteoblastoma accounts for 1% of all bone tumors and 3% of all benign bone tumors, worldwide (4). Osteoblastoma most frequently occurs in young adults and primarily involves the vertebral column, long bones, small bones of the extremities and facial bones, including the jaw (4–6). The first case of osteoblastoma described in the jaw bones was reported by Borello and Sedano in 1967 (7). Subsequent data has revealed that the mandibular bones of the jaw are more commonly affected by osteoblastoma than the maxillary bones, with the majority of osteoblastoma affecting the mandibular posterior region (8). Osteoblastoma can be classified into two major clinicopathological forms as follows: The benign form, which has a slow growth rate, a well-defined sclerotic margin and is moderately well vascularized with a mild inflammatory response; and the aggressive form, which exhibits locally aggressive behavior with a tendency to recur, often complicating its differentiation from low-grade osteosarcoma (9). Osteoblastoma may have a different clinical prognosis depending on its propensity to recur, its locally aggressive behavior and, in rare cases, whether malignant transformation takes place (5). Morphologically, osteoblastoma is identified by osteoid and woven bone deposition and enriched osteoblasts, which are frequently in close association with newly formed bone (4). Therefore, evaluation of clinical, histological and radiological findings is essential for the definitive diagnosis and effective treatment of osteoblastoma (6).

The current study reports a rare case of benign osteoblastoma in a 12-year-old female patient, involving the right mandible, which was treated by surgical excision. Written informed consent was obtained from the patient’s family.

## Case report

**Figure 1:**
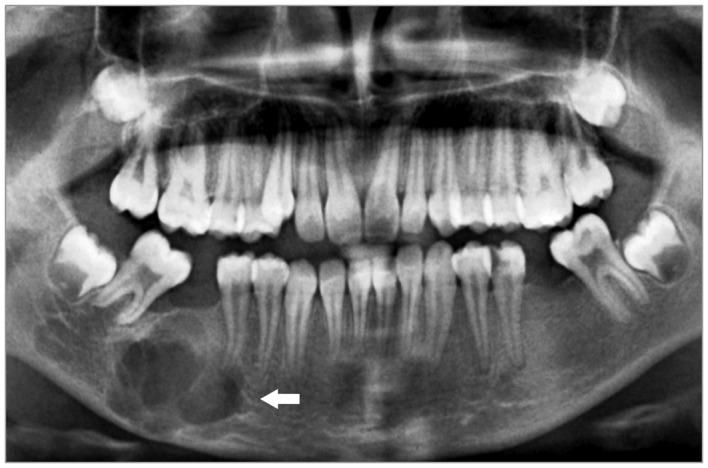
Radiograph indicating the 3.5 cm lesion (arrow) in the body of the right mandible.

**Figure 2:**
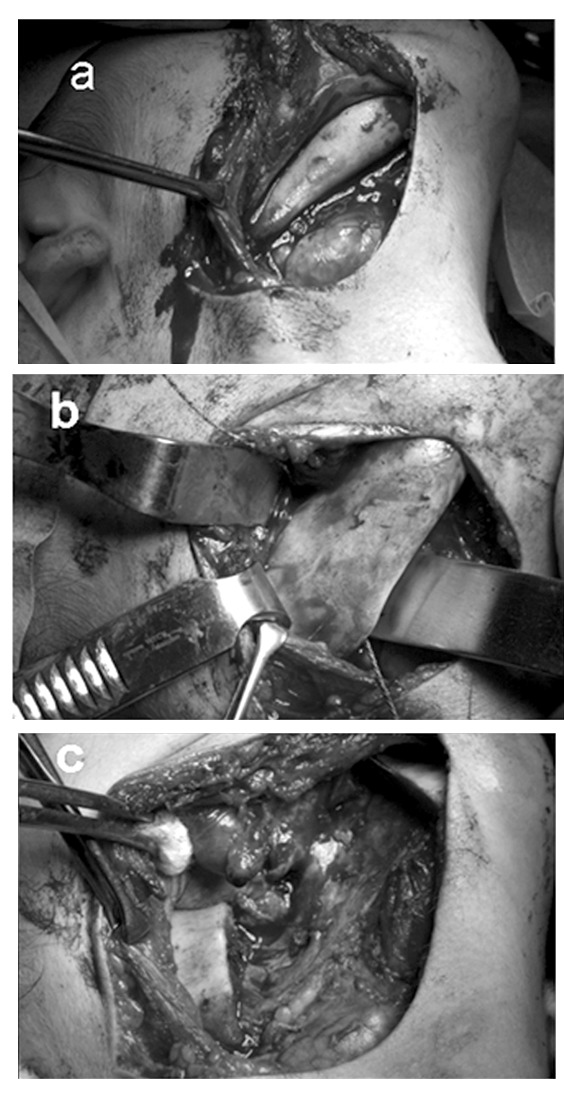
Surgical procedure for the osteoblastoma resection. (A) Localization of the tumor; (B) resection of the lesion at the angle of the mandible and (C) defect of the lower jaw following surgery.

**Figure 3:**
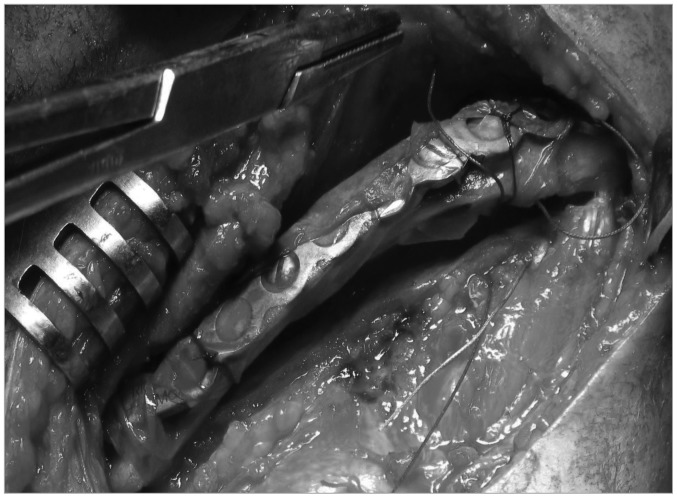
(A) Reconstruction of the jaw defect with a titanium implant and an autologous VI rib graft.

**Figure 4:**
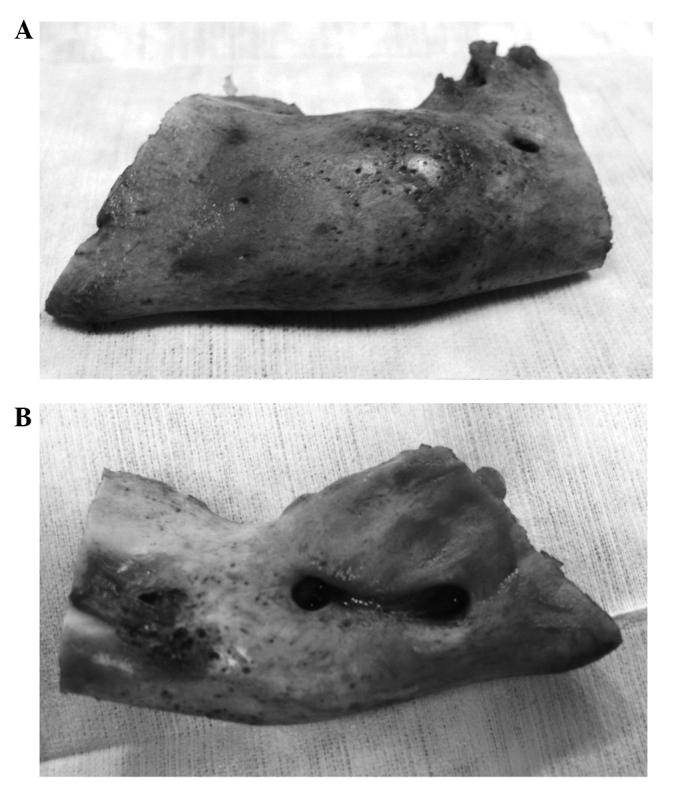
(A) Macroscopic view of the resected bone (outer surface) and (E) macroscopic view of the resected bone (inner surface).

**Figure 5:**
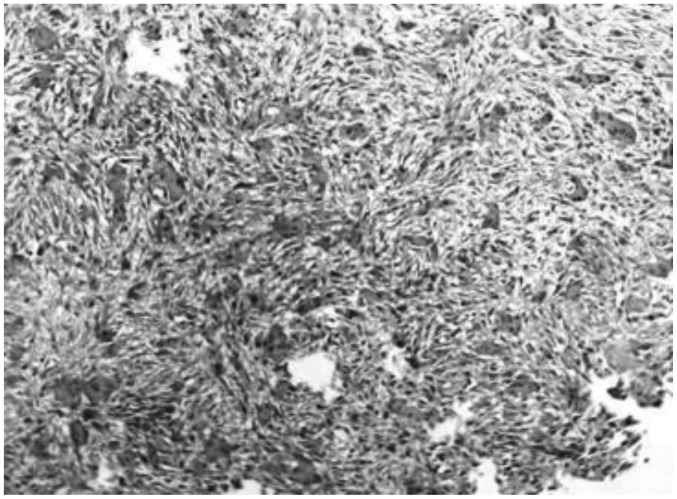
Histopathological image of the lesion (hematoxylin and eosin staining; magnification, ×40).

**Figure 6:**
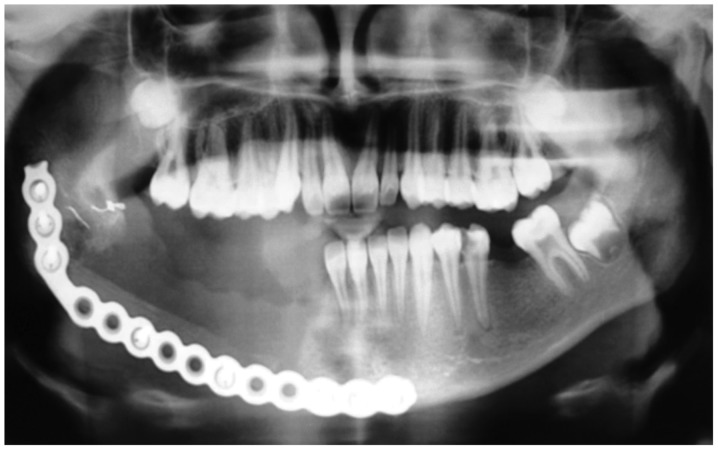
Radiograph of the reconstructed jaw defect at the five-month follow-up.

A 12-year-old female patient, reported to the Department of Oncology, Tbilisi State Medical University (Tbilisi, Georgia) in July 2013 with the complaint of intraoral swelling and pain on the right side of the lower jaw. A clinical examination revealed the presence of a firm, palpable tumor mass, the majority of which was situated within the body of the right mandible. Radiological imaging revealed that the lesion consisted of regular contours and exhibited a marginal amount of calcification (Fig. 1). Due to consideration of the patient history, clinical examination and the nature of the growth, a clinical diagnosis of a benign tumor of the bone was determined. Thus, the patient was subjected to a right lower jaw partial resection and the lesion was completely surgically removed (Fig. 2A–C). The jaw defect was reconstructed using titanium implants and an autologous VI rib graft (Fig. 3). Upon macroscopic examination, the excised tumor was observed to be an oval mass measuring 3.5 cm at its greatest diameter, with a brown, nodular outer surface (Fig. 4) and predominantly red cut surface. The tumor had a rough consistency, with soft and firm areas of bone tissue. The specimen was sent for histopathological analysis, which revealed vascularized fibrous connective tissue with bony trabeculae, which was lined by numerous osteoblasts and scattered osteoclasts (Fig. 5). Based on the histomorphologic characteristics of the tumor, clinical observation and radiological evidence, the diagnosis of a benign osteoblastoma of the mandible was established. During surgery, it was noted that the lesion had not extended to the resection margin, indicating the complete removal of the tumor. This is supported by continued patient follow-up, with the patient feeling generally healthy and radiograph images indicating no signs of recurrence or complication five months subsequent to the surgery (Fig. 6).

## Discussion

Osteoblastoma is an osteoid and bone-forming benign tumor of the bone. Jaffe and Lichtenstein (2,3) were the first to propose that osteoblastoma is a true neoplasm of osteoblastic origin, however, other studies hypothesized that osteoblastoma arise as a result of trauma or inflammation (10). The age range of patients that present with the disease is 5–78 years, although it typically occurs in the second and third decades of life (11,12). Furthermore, the incidence rate is higher in males than in females, with a ratio of 3-2:1 (5,11,13). Osteoblastoma can arise in any bone in the body, however, it rarely involves the maxilla and mandible. The mandible is affected more commonly than the maxilla, with the majority of lesions occurring in the mandible body (14). In addition to being characterized as benign or aggressive, osteoblastoma growth can be classified as cortical, medullary, or periosteal. Whilst cortical growth commonly occurs in extragnathic craniofacial bones, it does not occur in the jaw (9). Clinically, osteoblastoma may be associated with painful or painless characteristics.

Due to the rarity and non-specific manifestation of osteoblastoma, diagnosis can be challenging. Radiological imaging may vary depending on the size of the tumor and the intensity of the calcification, although osteoblastomas commonly form well-circumscribed, fully radiolucent or calcified lesions ≤12 cm in diameter (15). Osteoblastoma must be differentiated from other bone-producing lesions, such as osteoid osteoma, osteosarcoma, cementoblastoma and ossifying fibroma as this may lead to improved treatment and prognosis (16). However, the differential diagnosis may be complicated due to the tumor’s rarity, ambiguous clinicoradiological presentation, and histopathologic features, which occasionally resemble osteosarcoma in particular. Histological analysis of osteosarcoma reveals pleomorphic osteoblasts and osteocytes, and malignant stromal cells. In benign osteoblastoma, stromal connective tissue cells do not demonstrate sarcomatous growth, and mitoses and sarcoma giant cells are absent. Furthermore, the cells that are enmeshed in the osteoid matrix are relatively small and homogeneous (8,17).

Conventional osteoblastomas are biologically benign with limited growth potential, typically ≤4 cm in diameter. However, a small subgroup of osteoblastomas possess a locally aggressive growth pattern and are usually >4 cm in diameter. These tumors are distinct from conventional osteoblastoma and are classified as osteoblastoma-like osteosarcomas, malignant osteoblastomas or aggressive osteoblastomas (18). It is hypothesized that, when compared with benign osteoblastoma, aggressive osteoblastomas occur in older patients and demonstrate clinically aggressive behavior. Aggressive osteoblastomas invade adjacent tissues and recur in 10–21% of patients, however, do not metastasize. Certain authors advocate that lesions described as aggressive osteoblastoma are actually well-differentiated osteosarcomas resembling osteoblastomas. Therefore, the diagnostic evaluation is based on the histologic features and the clinical behavior of the lesion (11).

Additionally, in view of the supposed benign nature of osteoblastoma tumors, surgical excision is the treatment of choice. Since recurrence is a rare event and usually attributable to an incomplete excision, the overall prognosis for osteoblastoma patients is considered to be good (4).

In conclusion, in the case of the present study, the clinical presentation of the disease was rare, however, the histopathologic findings were in accordance with those reported in previous literature. Correct diagnosis and complete surgical excision of the tumor is essential to minimize the risk of recurrence and the potential malignization of a benign osteoblastoma.
